# High L-Carnitine Levels Impede Viral Control in Chronic Hepatitis B Virus Infection

**DOI:** 10.3389/fimmu.2021.649197

**Published:** 2021-06-21

**Authors:** Shuqin Gu, Xin Fu, Guofu Ye, Chengcong Chen, Xiaoyi Li, Shihong Zhong, Libo Tang, Haitao Chen, Deke Jiang, Jinlin Hou, Yongyin Li

**Affiliations:** State Key Laboratory of Organ Failure Research, Guangdong Provincial Key Laboratory of Viral Hepatitis Research, Department of Infectious Diseases, Nanfang Hospital, Southern Medical University, Guangzhou, China

**Keywords:** L-carnitine, hepatitis B virus, immunosuppression, immune cells, single-nucleotide polymorphism

## Abstract

Persistent antigen exposure during chronic hepatitis B infection leads to exhausted immune responses, thus impeding viral control. In recent years, immunometabolism opens new therapeutic possibilities for the modulation of immune responses. Herein, we investigated the immunomodulatory effect of L-carnitine (L-Cn) on immune cells in chronic HBV infection. In this study, 141 treatment-naïve patients with chronic HBV infection, 38 patients who achieved HBsAg loss following antiviral treatment, and 47 patients who suffered from HBV-related HCC from real-life clinical practice were recruited. The plasma L-Cn levels were measured by ELISA. RNA sequencing was conducted to define the transcriptional profiles of peripheral blood mononuclear cells after L-Cn stimulation. *In vitro* assays were performed to assess the effect of L-Cn on immune cells; the frequencies and function of immune cells were analyzed by flow cytometry. We found that compared with patients with HBsAg loss, patients with HBsAg positivity and patients who suffered from HBV-related HCC had higher levels of L-Cn, and the plasma levels of L-Cn in the HBeAg-positive chronic hepatitis patients who had elevated ALT were significantly higher than that of HBeAg-negative chronic infection and HBsAg loss groups. Moreover, a positive correlation between plasma levels of L-Cn and HBsAg levels was found. Additionally, RNA sequencing analysis demonstrated that L-Cn altered the transcriptional profiles related to immune response. *In vitro* assays revealed that L-Cn suppressed the proliferation of and IFN-γ production by CD4^+^ and CD8^+^ T cells. It also down-regulated the proliferation and IgG production of B cells. Notably, L-Cn enhanced IL-10 secretion from regulatory T cells and up-regulated the expression of inhibitory receptors on T cells. Moreover, a variant in *CPT2* (rs1799821) was confirmed to be associated with L-Cn levels as well as complete response in CHB patients following Peg-IFNα antiviral therapy. Taken together, the immunosuppressive properties of L-Cn may hinder the control of HBV in chronic HBV infection, implicating that L-Cn manipulation might influence the prognosis of patients with HBV infection.

## Introduction

Chronic hepatitis B virus (HBV) infection remains a serious public health issue, which is the leading cause of chronic hepatitis, cirrhosis, and hepatocellular carcinoma (HCC) (1). Nucleos(t)ide-analogues are currently effective at inhibiting HBV replication, and interferon (IFN) therapy can convert an activity into an inactive infection. However, these treatment strategies could not eliminate HBV because of the stability of covalently closed circular DNA (cccDNA) in infected hepatocytes (2). An achievement of HBsAg loss represents the hallmark of resolution of HBV infection and is a therapeutic goal for a functional cure; unfortunately, this optimal outcome is rarely achieved (3, 4). Therefore, investigating the underlying factors that go against the achievement of HBsAg loss during chronic HBV infection is warranted.

The disorder of T cells mediated cellular immune response favored viral persistence and pathogenesis of hepatitis B (5). Inducing powerful HBV-specific CD4^+^ and CD8^+^ T cell responses and the production of HBV-related antibodies determine the outcome of HBV infection. Recently, cumulative data from experimental and clinical studies have highlighted the critical role of the metabolic milieu in regulating host immunity (6–10). Immune response requires distinct metabolic programs to support immune cell survival, development, differentiation, fate, and behavior. L-carnitine (L-Cn) is essential to transport the chains of fatty acids into the mitochondria for β oxidation. The carnitine pool consists of free carnitine and acylcarnitine esters, which were bounded to different fatty acids. In individuals, about 75% of L-Cn is obtained from the diet, and only 25% of it comes from endogenous biosynthesis with lysine and methionine in the kidney, liver, and brain; additionally, carnitine is not metabolized but is excreted as free carnitine in urine. L-Cn is the exclusively physiologically active form of free carnitine (11). A previous study has documented an association between lower baseline levels of plasma L-Cn and HBsAg loss in chronic hepatitis B (CHB) patients receiving Peg-IFNα based treatment (12). Furthermore, the report demonstrated that L-Cn limited the proliferation of HBV-specific CD8^+^ T cells. Besides, it has been shown that L-Cn displays immune suppressive properties in Crohn’s disease (13, 14). On the contrary, previous studies demonstrated the protective effect of carnitine supplementation on ALT normalization in patients with CHB when combined with entecavir, improvement in sarcopenia and hyperammonemia in patients with liver cirrhosis, and hospital admissions reduction in patients with hepatic encephalopathy (15–18). However, available findings regarding plasma L-Cn levels in patients with chronic HBV infection remain somewhat contradictory, and the effect of L-Cn on the expression of inhibitory receptors and the production of suppressive cytokines by immune cells have not yet been fully elucidated. In the present study, we aimed to investigate L-Cn profiles in patients with different statuses of chronic HBV infection as well as the immunomodulatory effect of L-Cn on immune cells *in vitro*, and factors that affect plasma L-Cn levels were also primarily explored.

## Materials And Methods

### Study Subjects

A total of 179 chronic HBV infection patients were enrolled for a cross-sectional study. One hundred and forty-one treatment-naïve patients were classified into hepatitis B e antigen-negative chronic infection (HBeAg^-^CInf, n=44), HBeAg-negative chronic hepatitis (HBeAg^-^CHep, n=20), HBeAg^+^CInf (n=36), and HBeAg^+^CHep (n=41) based on an issued clinical practice guideline (4). Thirty-eight CHB patients who achieved HBsAg loss (n=38) following antiviral treatment and 47 patients who suffered from HBV-related HCC were recruited. Another 34 HBsAg-negative healthy individuals with normal alanine aminotransferase (ALT) levels were enrolled as healthy controls (HCs) for the cross-sectional study. Additionally, 648 HBeAg-positive CHB patients who treated with Peg-IFNα at the dose of 180 µg/week for 48 weeks and a treatment-free follow-up for 24 weeks (Peg-IFNα-2a, n=324; Peg-IFNα-2b, n=324) were used to evaluate the association of single-nucleotide polymorphism (SNP) rs1799821 genotype with complete response (CR, defined as HBeAg seroconversion and an HBV DNA level < 2000 IU/mL at week 72). Details of patients were previously reported (19, 20). Patients suffering from autoimmune diseases, other active diseases, or coinfection with HAV, HCV, HDV, or HIV were excluded. All subjects were recruited at Nanfang Hospital (Guangzhou, China). This study was conducted in compliance with the Declaration of Helsinki and was approved by the Ethical Committee of Nanfang Hospital. A written informed consent form was obtained from all participants.

### Serological Assays and HBV DNA Assays

The levels of human serum HBsAg, HBsAb, HBeAg, and HBeAb were quantitatively determined using the Roche COBAS^®^ 6000 analyzer (Roche Molecular Diagnostics, Rotkreuz, Switzerland). Levels of serum HBV DNA were quantified by the Roche LightCycler^®^ 480 II (Roche Molecular Diagnostics, Pleasanton, CA) with Hepatitis B Viral Quantitative Fluorescence Diagnostic Kit (Sansure Biotech, Hunan, China). The lower limit of detection of HBsAg is < 0.05 IU/mL. The detection limit of HBV DNA is no lower than 100 IU/mL. The normal ranges for ALT and AST levels were 9-50 U/L and 15-40 U/L, respectively.

### Enzyme-Linked Immunosorbent Assay (ELISA)

Plasma was immediately withdrawn and frozen at -20°C until use. Plasma L-Cn levels were measured using one specific ELISA kit (CUSABIA, Wuhan, China), and the concentration of Immunoglobulin G (IgG) of cultured supernatant was quantified in duplicate wells by commercial IgG ELISA kits (Invitrogen) according to the manufacturer’s instructions.

### RNA-Sequencing and Data Analysis

Peripheral blood mononuclear cells (PBMCs) were isolated from fresh heparinized blood by Ficoll-Hypaque density gradient centrifugation, and some cells were cryopreserved in liquid nitrogen for further analysis. Fresh PBMCs were obtained from 3 treatment-naïve patients with chronic HBV infection and then were stimulated with L-Cn (20 mg/mL) or not for 3 days in an incubator at 37°C supplied with 5% CO_2_. Total RNA was directly extracted using TRIzol reagent (Life Technologies, Foster City, CA, USA) and subjected to library construction and deep sequencing on Illumina HiSeq™ 2500 by Gene Denovo Biotechnology Co., Ltd (Guangzhou, China). Gene expression levels were normalized based on the fragments per kilobase million reads (FPKM). Fold changes were calculated for all possible comparisons and a two-fold cutoff was used to select genes with expression changes. All raw transcriptome data have been deposited in NCBI (SRA accession number: PRJNA673447, https://www.ncbi.nlm.nih.gov/sra/PRJNA673447). Gene Ontology (GO), Kyoto Encyclopedia of Genes and Genomes (KEGG), and Reactome pathway analysis were performed by Guangzhou Gene Denovo Biotechnology Co., Ltd.

### Cell Sorting

Fresh isolated PBMCs were stained with anti-CD4-PE-Cy7, anti-CD8-APC, and anti-CD19-PE. The cell suspension was sorted by a BD Aria III flow cytometer (BD Bioscience). Cell purity was > 99%.

### Proliferation Assay

Fresh isolated PBMCs or purified T/B cells labeled carboxyfluorescein succinimidyl ester (CFSE) were cultured with different concentration of L-Cn (Sigma-Aldrich) for 5 days in the presence of soluble anti-CD3 (5 µg/mL, Biolegend) and anti-CD28 (5 µg/mL, Biolegend) for T cell proliferation assay or 7 days in the presence of CPG (10 µg/mL, InvivoGen) for B cell proliferation assay or with medium only as control. At the end of the culture, cells were harvested, stained with anti-CD4-PE-Cy7, anti-CD8-APC, or anti-CD19-PE, and were analyzed by a BD Canto II flow cytometer. Dead cells were excluded using Live/Dead staining (Life Technologies). The proliferation rate of each cell subset was expressed as the percentage of cells that diluted CFSE intensity at least once.

### Phenotype Analysis and Intracellular Cytokine Staining (ICS)

Fresh isolated human PBMCs after stimulating with or without L-Cn (20 mg/mL) for 72 h in the presence of anti-CD3 (5 µg/mL, Biolegend) and anti-CD28 (5 µg/mL, Biolegend) were stained with fluorescence phenotype antibodies at 4°C for 30 minutes and analyzed on a BD FACS-Canto II or Aria III flow cytometer (BD Bioscience). To assess the effect of L-Cn on T cell subsets, PBMCs or purified T cells were stimulated with or without L-Cn (20 mg/mL) in the presence of anti-CD3/CD28 (5 µg/mL) for 72 h and restimulated with or without L-Cn for another 6 h in the presence of PMA (50 ng/mL), ionomycin (0.75 µg/mL), and BFA (1 µg/mL). The ICS was performed as previously described (21). Briefly, cells stained with the indicated antibodies (**Supplementary Table 1**) and then fixed and permeabilized using a Cytofix/Cytoperm kit (BD Bioscience) or a Transcription Factor Buffer Set (BD Biosciences) and stained with the corresponding antibodies (IFN-γ, IL-21, IL-10, FoxP3, TCF1, and Eomes). All flow cytometric analysis was performed using FlowJo V10.0.7 software (Treestar).

### *In Vitro* Antibody Production and Enzyme-Linked Immunospot Assay (ELISPOT)

To assess the effect of L-Cn on B cells, purified CD19^+^ B cells were cultured with or without L-Cn in the presence of CPG (2.5 µg/mL, InvivoGen) and PWM (5 µg/mL, Sigma-Aldrich) for 13 days. The frequency of IgG-secreting B cells was determined using the ELISPOT assay. Briefly, PBMCs (5 × 10^5^ cells/well) were cultured in 200 uL RPMI-1640 medium/10%FBS containing R848 (1 µg/mL) and rIL2 (10 ng/mL, Mabtech, Nacka Strand, Sweden) with or without L-Cn (10 mg/mL) for 5 days in a round-bottomed 96-well plate. Sterile ELISPOT plates with a PVDF membrane (Millipore Corp, Bedford, MA, USA) were coated with anti-human IgG (15 µg/mL, Mabtech, Nacka Strand, Sweden) overnight at 4°C. After 5 days, the cells were transferred to ELISPOT plates and incubated with or without L-Cn for an additional 24 h. The plates were subsequently washed and incubated with biotin-labeled anti-IgG mAb (Mabtech, Sweden) and horseradish peroxidase-conjugated streptavidin (Mabtech, Nacka Strand, Sweden). Spots were counted by Immuno-SpotS6 Ultra Analyzer (Cellular Technology, Inc., Santa Monica, CA).

### GEO Dataset

We analyzed the microarray data from the Gene Expression Omnibus series GSE62595 (https://www.ncbi.nlm.nih.gov/geo/query/acc.cgi?acc=GSE62595), which provides gene expression data from L-Cn-treated *in vivo* bovine embryos (Jersey and Holstein breeds, 4 L-Cn treated and 4 controls for each). *IL-21*, *IFNG*, *CD86*, *CD33*, *Arg1*, *CD8b*, *PD-L1*, *CTLA4*, and *HAVCR2* gene expression levels in the L-Cn-treated and control groups were analyzed in this study.

### DNA Extraction and Genotyping

Genomic DNA was extracted from PBMCs of patients using a TIANamp Blood DNA Kit (Tiangen, Beijing, China). The selected 7 SNPs in *ALX3*, *CPT1B*, *CPT2*, *DMGDH*, *PEX5L*, and *SLC22A4* were genotyped using an improved multiplex polymerase chain reaction-ligation detection reaction (LDR) technique with technical support from Genesky Biotechnology Inc. (Shanghai, China). DNA sequencing was used to validate the genotyping by LDR. Results of LDR corresponded with the results of sequencing for the randomly selected DNA samples from each genotype. Genotyping of rs1799821 in patients treated with Peg-IFNα was performed as previously described (20).

### Statistical Analysis

All data are expressed as median (interquartile range). Continuous variables were compared by Mann-Whitney’s *U* test or Wilcoxon sighed-rank test when two groups were compared. Kruskal-Wallis H test was used when more than two groups were compared, Friedman test was used when more than two repeated measures were compared, performing a *post hoc* test (Dunn’s test) that applies correction for multiple comparisons. Correlations between variables were assessed with Spearman’s rank-order correlation coefficient. Categorical variables were compared by the Chi-square test. Multiple logistic regression analyses were used to adjust the confounding effect of baseline variables. SPSS Statistics 20.0 (Chicago, IL) and the GraphPad Prism 7 software were used for statistical analysis. All the tests were two-sides, and a *P* value < 0.05 was considered statistically significant.

## Results

### Plasma L-Cn Levels Are Elevated in Chronic HBV Infection and HBV-Related HCC but Decreased in HBsAg Loss

**Figure 1:**
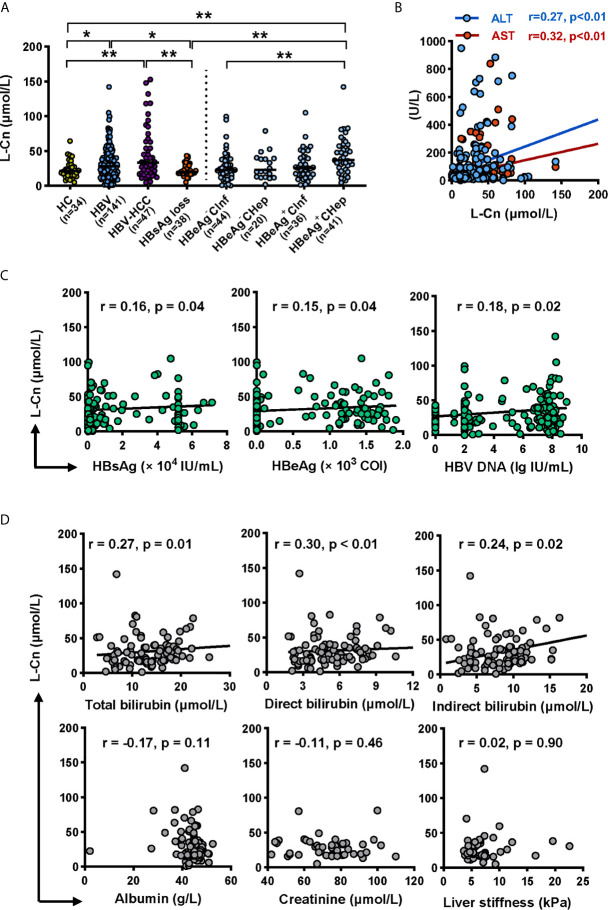
Analyses of the L-Cn levels in cross-sectional cohorts. **(A)** Comparing plasma L-Cn levels within patients with chronic HBV infection, HCC, HBsAg loss, and HCs. **(B)** The correlation between the plasma L-Cn levels and serum levels of ALT or AST. **(C)** Spearman correlation between the levels of plasma L-Cn and the levels of serum virological parameters. **(D)** Spearman’s correlation between plasma L-Cn levels and the levels of serum biochemical parameters and the value of liver stiffness. **(A)** Mann-Whitney *U* test or Kruskal-Wallis H test and Dunn’s multiple comparisons test. **(B–D)** Spearman’s rank correlation test. ^*^ *P* < 0.05, ^**^ *P* < 0.01. ALT, alanine aminotransferase; AST, aspartate aminotransferase; HCC, hepatocellular carcinoma; HCs, healthy controls; L-Cn, L-carnitine.

Firstly, we attempted to evaluate the plasma L-Cn status of patients with chronic HBV infection (**Table 1**). A higher L-Cn level was observed in patients with chronic HBV infection (excluding HBsAg loss patients) or HCC, relative to HCs; moreover, L-Cn levels of patients with chronic HBV infection or HCC were higher than those who achieved HBsAg loss (**Figure 1A**). The plasma level of L-Cn in the HBeAg^+^CHep group who had flared ALT was significantly elevated than the HBeAg^-^CInf, HCs, and HBsAg loss groups (**Figure 1A**). We next examined whether plasma L-Cn levels were correlated or not with serum biochemical and virological parameters. A positive correlation between plasma levels of L-Cn and serum levels of ALT and AST was found (**Figure 1B**). Notably, such positive correlations were also observed between plasma L-Cn levels and HBsAg levels as well as HBeAg and HBV DNA levels (**Figure 1C**). Further analysis revealed that plasma L-Cn levels were positively correlated with serum levels of total bilirubin, direct bilirubin, and indirect bilirubin, but not with the serum albumin levels, creatinine concentration, and liver stiffness (**Figure 1D**). Together, these results indicated that persistent HBV infection might act on L-Cn metabolism and plasma levels of L-Cn appear to be associated with the prognosis of chronic HBV infection.

**Table 1:** Clinical characteristics of the study subjects.

Group	Healthy controls	HBeAg- chronic infection	HBeAg- chronic hepatitis	HBeAg+ chronic infection	HBeAg+ chronic hepatitis	HBsAg loss	HBV-HCC
No. of patients (M/F)	34 (20/14)	44 (22/22)	20 (18/2)	36 (20/16)	41 (24/17)	38 (33/5)	47 (44/3)
Age (year)	24.00 (23.00-28.25)	35.00 (31.00-43.00)	34.00 (26.00-43.00)	28.00 (25.00-33.00)	27.00 (24.00-32.50)	40.00 (34.00-51.50)	53.00 (44.00-62.00)
HBV DNA (lg IU/mL)	n.d.	2.00 (2.00^a^-2.28)	5.99 (4.87-6.95)	7.80 (7.65-8.11^b^)	7.74 (7.08-8.06^b^)	T.N.D	4.01 (0.00^c^-5.79)
ALT (ULN)	0.29 (0.26-0.36)	0.40 (0.30-0.54)	3.35 (1.88-13.00)	0.52 (0.36-0.62)	3.08 (1.94-6.38)	0.48 (0.30-0.64)	0.66 (0.50-1.06)
AST (ULN)	0.48 (0.40-0.53)	0.50 (0.43-0.60)	2.21 (1.33-7.13)	0.53 (0.48-0.58)	1.98 (1.25-4.96)	0.55 (0.45-0.65)	1.28 (0.70-2.50)
HBsAg positive	0	44	20	36	41	0	46
HBsAb positive	33	0	0	0	0	19	1
HBeAg positive	0	0	0	36	41	2	9
HBeAb positive	0	44	20	0	2	23	35
Indirect bilirubin (μmol/L)	n.d	6.10 (3.90-9.00)	7.95 (4.03-10.35)	7.60 (6.15-9.10)	8.20 (5.20-11.43)	9.50 (7.35-11.03)	10.40 (6.40-15.90)
Direct bilirubin (μmol/L)	n.d	4.30 (2.60-5.60)	5.50 (3.48-9.15)	4.20 (3.40-6.00)	5.40 (3.58-7.43)	5.70 (3.68-7.63)	9.00 (5.20-18.00)
Total bilirubin (μmol/L)	n.d	9.80 (6.40-13.90)	13.45 (7.5-19.50)	11.80 (9.75-15.60)	14.25 (8.95-19.65)	14.95 (11.78-18.40)	18.10 (12.20-41.10)
L-Cn (μmol/L)	21.01 (14.19-27.85)	22.47 (12.69-38.26)	24.89 (12.79-40.24)	25.48 (16.34-41.72)	36.45 (24.48-59.26)	19.65 (17.11-29.59)	33.28 (18.14-63.41)

Data were shown as median (25-75% percentile).

a Twenty-eight subjects were lower than 2.0 in HBeAg- chronic infection.

b Two/Three subjects were higher than 8.2 in HBeAg+ chronic infection/hepatitis.

c Fifteen subjects were T.N.D in HBV-HCC.

ALT, alanine aminotransferase; AST, aspartate aminotransferase; HBeAb, hepatitis B e antibody; HBeAg, hepatitis B e antigen; HBsAb, hepatitis B surface antibody; HBsAg, hepatitis B surface antigen; HCC, hepatocellular carcinoma; L-Cn, L-carnitine; n.d., not determined; T.N.D, target not detected; ULN, the upper limit of normal.

### L-Cn Altered the Landscape of Gene Expression Profiles of PBMCs in Chronic HBV Infection

**Figure 2:**
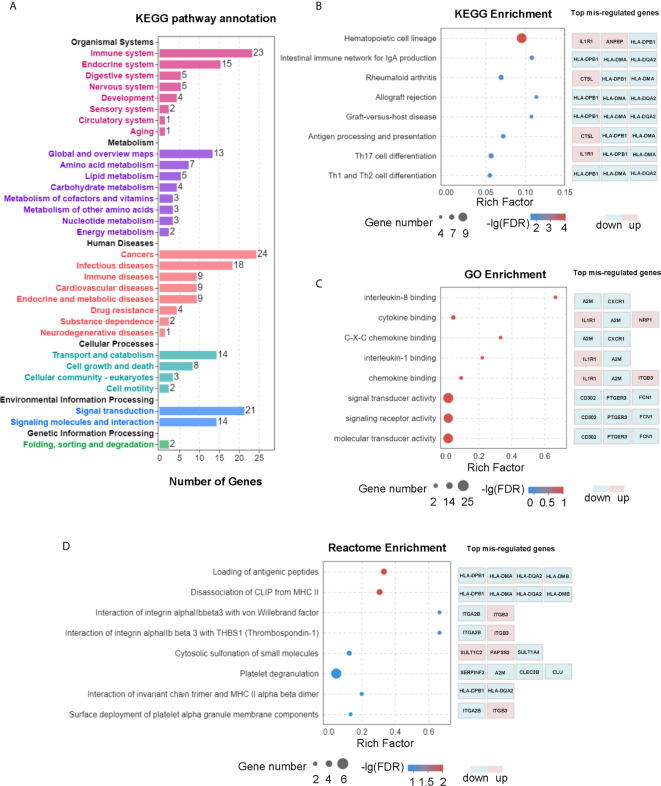
Transcriptomic analysis revealed multiple changes in PBMCs after L-Cn stimulation. **(A)** KEGG analysis of genes enriched based on 168 differentially expressed genes with a fold change ≥ 2, and *P* < 0.05 showed changes in gene sets. **(B)** KEGG analysis was carried out to identify the top 20 differential pathway enrichment between L-Cn and the control group. **(C)** The top 20 statistics of GO terms of molecular function were plotted after L-Cn stimulation. **(D)** Reactome enrichment analysis identified the top 20 differential pathways. Rich factor refers to the ratio of the number of genes differentially expressed in the pathway entry to the total number of genes in the pathway entry. Eight of the top 20 differential pathways were shown.

To uncover the molecular mechanism of L-Cn that may be involved in reprogramming PBMCs after L-Cn stimulation, transcriptome profiling by RNA-Sequencing was conducted. KEGG pathway enrichment analysis based on a subset of 168 genes that differentially expressed in the presence of L-Cn revealed changes in the gene sets related to metabolism, cellular processes, and information processing of PBMCs, particularly in the immune system, cancers, and infectious diseases (**Figure 2A**). Pathways of antigen processing and presentation, Th17 cell differentiation, and Th1 and Th2 cell differentiation were significantly altered in the setting of exposure to L-Cn (**Figure 2B**). In addition, the GO analysis of genes enriched in terms of molecular function showed a prominent abundance of gene signatures associated with cytokine binding and C-X-C chemokine binding (**Figure 2C**). Further analysis by Reactome Pathway Database showed significant enrichment of differentially expressed genes associated with loading of antigenic peptides and disassociation of CLIP from MHC II (**Figure 2D**). These changes suggested L-Cn triggers multiple metabolic reprogramming and functional reinventing on immune cells.

### Excess L-Carnitine Impairs Immune Responses Against HBV

**Figure 3:**
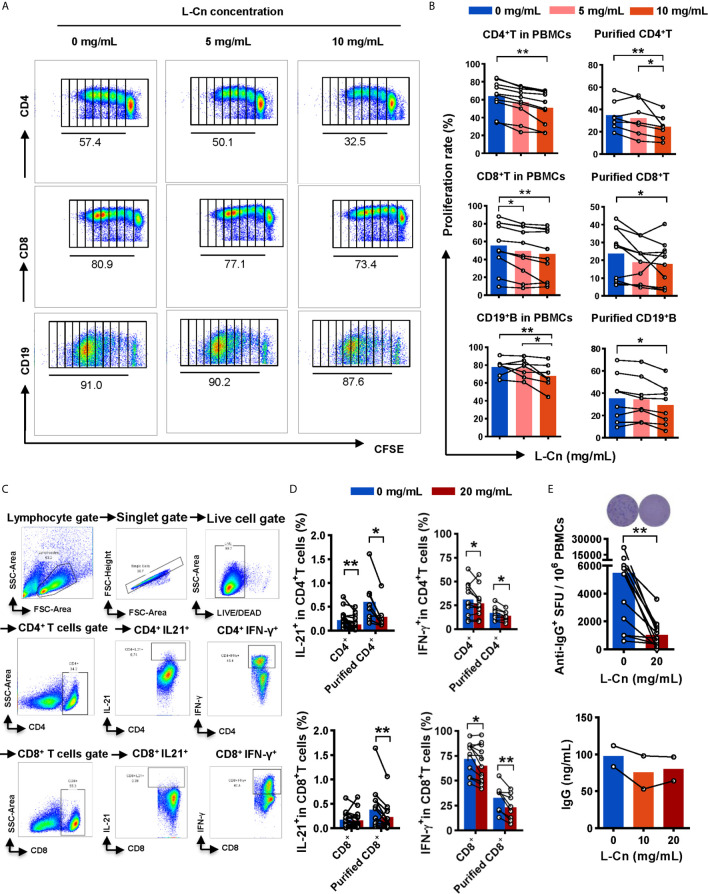
The effect of L-Cn on immune cells. **(A, B)** The proliferative response of CD4^+^ T cells, CD8^+^ T cells, and CD19^+^ B cells to L-Cn stimulation. Different levels of L-Cn were used to coculture with PBMCs or sorted cell subsets. **(C)** Gating strategy. **(D)** IFN-γ and IL-21 production of T cells or purified T cell subsets stimulated with L-Cn (20 mg/mL) in the presence of anti-CD3/28 and PMA/ionomycin. PBMCs or purified T cells were stimulated with or without L-Cn (20 mg/mL) in the presence of anti-CD3/CD28 (5 µg/mL) for 72 h and restimulated with or without L-Cn for another 6 h in the presence of PMA (50 ng/mL), ionomycin (0.75 µg/mL), and BFA (1 µg/mL) and subsequently intracellular staining was performed and then was analyzed on a BD FACS-Canto II flow cytometer. **(E)** Detection of IgG-secreting B cells by ELISPOT assay and measurement of IgG concentration in the supernatant by ELISA assay. Image showing the results of representative ELISPOT assay. **(B)** Friedman test and Dunn’s multiple comparisons test. **(D, E)** Wilcoxon signed-rank test. ^*^ *P* < 0.05, ^**^ *P* < 0.01. IgG, Immunoglobulin G; L-Cn, L-carnitine; SFU, spot-forming units.

Next, the influence of L-Cn on immune cells against HBV was explored. PBMCs or purified T/B cells were cultured in the presence or not of L-Cn *in vitro*. Relative to untreated cells, we found that L-Cn treatment on PBMCs significantly inhibited the proliferation of CD4^+^ T cells and CD8^+^ T cells in a dose-dependent manner (**Figures 3A, B** **)**. Given that PBMCs contain a mixture of cell types, the effect of L-Cn was assessed on the sorted immune cell subsets. L-Cn stimulation (10 mg/mL) led to a decrease in the proliferation of purified CD4^+^ T cells and CD8^+^ T cells (**Figure 3B**). A similar reduction in proliferation of CD19^+^ B cells in PBMCs or purified B cells was observed (**Figures 3A, B** **)**. Strikingly, reduced production of IL-21 by CD4^+^ T cells, and decreased frequency of IFN-γ-expressing CD4^+^ T cells and CD8^+^ T cells were observed in the presence of L-Cn (**Figures 3C, D** **)**. Interestingly, exposure to L-Cn resulted in decreased production of both IFN-γ and IL-21 by purified CD4^+^ T cells and CD8^+^ T cells (**Figure 3D**). Notably, coculture with L-Cn resulted in significantly reduced frequencies of IgG-secreting B cells and a tendency of reduced production of IgG in the supernatant by purified CD19^+^ B cells, relative to the control group (**Figure 3E**). These findings collectively suggested that excess L-Cn abrogates T-cell and B-cell response, which might impede the achievement of HBsAg loss in chronic HBV infection.

### L-Cn Promotes IL-10 Production by Treg and the Expression of Inhibitory Receptors on T Cell Subsets

**Figure 4:**
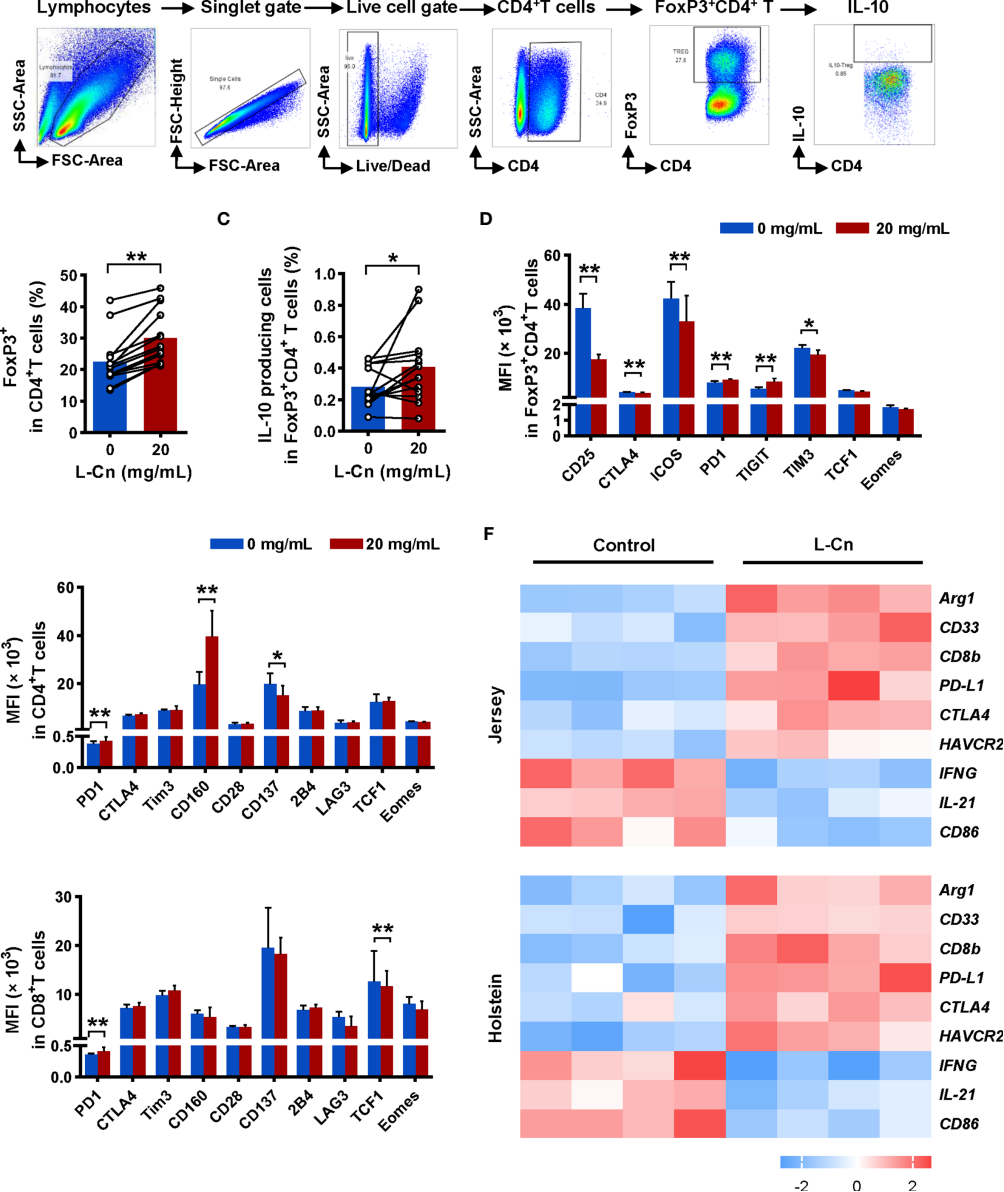
L-Cn up-regulates inhibitory receptors on T cells and enhances IL-10-producing Treg cells. **(A)** Gating strategy for FoxP3^+^CD4^+^ T cells. **(B, C)** The frequencies change and function of FoxP3^+^CD4^+^ regulatory T (Treg) cells after L-Cn stimulation. **(D, E)** The mean fluorescence intensity (MFI) of indicated activated and inhibitory receptors on T-cell subsets after L-Cn stimulation. **(F)** Heat map illustrating the relative expression of the indicated genes according to a GEO microarray dataset (GSE62595). **(B–E)** Wilcoxon signed-rank test. ^*^ *P* < 0.05, ^**^ *P* < 0.01. L-Cn, L-carnitine.

Subsequently, we explored the impact of L-Cn on FoxP3^+^CD4^+^ regulatory T (Treg) cells (**Figure 4A**). Strikingly, L-Cn up-regulated the frequency of Treg cells, along with the abundance of IL-10-producing Treg cells (**Figures 4B, C** **)**. We further characterized the expression of activated or inhibitory receptors on lymphocytes after L-Cn stimulation. We found that inhibitory receptors PD1 and TIGIT were dominantly increased on FoxP3^+^CD4^+^ Treg cells in L-Cn administration, while the expression of CD25, CTLA4, ICOS, and TIM3 were decreased (**Figure 4D**). CD4^+^ T cells showed an elevated intensity of PD1 and CD160 and declined intensity of CD137 after L-Cn treatment; meanwhile, an elevated intensity of PD-1 and a reduced intensity of transcriptional regulator TCF1 was observed on CD8^+^ T cells (**Figure 4E**). Next, to further verify the findings as mentioned above in our study, the transcriptional profiles were analyzed by using a GEO microarray dataset (GSE62595), which was derived from L-Cn-treated *in vivo* bovine embryos. As shown in **Figure 4F**, the transcriptional levels of *IL-21* and *IFNG* in the L-Cn-treated groups were significantly lower than those in the control groups in both Jersey and Holstein cattle, which were consistent with the results of the available *in vitro* experiments, while the transcripts encoding immunosuppressive profiles, such as myeloid-derived suppressor cells (*CD33*), arginase-1 (*Arg1*), and inhibitory receptors (*PD-L1*, *CTLA4*, and *HAVCR2*) were higher in the L-Cn-treated groups. These data indicate that L-Cn contributes to the production of suppressive cytokine and the expression of inhibitory receptors, thus resulting in the persistence of HBV infection.

### A Variant in *CPT2* (rs1799821) Is Associated With L-Cn Levels and the Complete Response of CHB Patients Treated With Peg-IFNα

**Figure 5:**
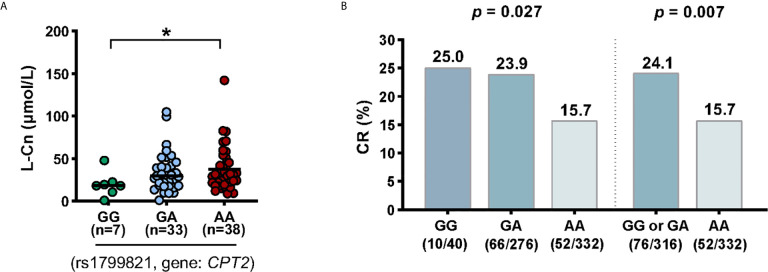
Association of *CPT2* rs1799821 with plasma L-Cn levels and CR in patients treated with Peg-IFNα. **(A)** Association of plasma L-Cn levels with SNP rs1799821 genotype. **(B)** Association of SNP rs1799821 genotype with CR in patients treated with Peg-IFNα. **(A)** Kruskal-Wallis H test and Dunn’s multiple comparisons test. **(B)** Chi-square test. ^*^ *P* < 0.05. CR, complete response.

Next, we tried to investigate the factors that influence the levels of plasma L-Cn. To this end, we analyzed the association between L-Cn and some reported SNPs (**Supplementary Figure 1**). We found that, for SNP rs179982 (gene *CPT2*), patients with GG genotype had lower plasma L-Cn levels than those with AA genotype (**Figure 5A**). Then, 648 HBeAg-positive CHB patients treated with Peg-IFNα were used to evaluate the association between rs1799821 genotype and complete response (**Table 2**). As shown in **Figure 5B**, after 48 weeks of therapy plus 24 weeks follow-up, 25.0%, 23.9%, and 15.7% of patients with rs1799821 GG, GA, and AA genotypes achieved CR, respectively. Patients carrying GG/GA genotypes displayed a higher rate of CR compared with those with AA genotype. Of note, there was a statistically significant association after adjustment for other covariates, such as gender, age, HBV genotype, baseline HBV DNA, HBsAg, HBeAg, and ALT levels (**Table 3**). These data implied that baseline L-Cn levels might be associated with the treatment response of CHB patients.

**Table 2:** Baseline clinical characteristics of patients treated with Peg-IFNα.

	CR (n=128)	NCR (n=520)	Overall (n=648)
Age (year), Mean (SD)	28.02 (6.04)	30.04 (6.71)	29.64 (6.63)
Male, N (%)	90 (70.31)	379 (73.03)	469 (72.49)
HBsAg, log_10_IU/mL; mean (SD)	4.17 (0.51)	4.31 (0.53)	4.28 (0.53)
HBeAg, log_10_PEIU/mL; mean (SD)	2.88 (0.47)	3.08 (0.57)	3.04 (0.56)
HBV DNA, log_10_IU/mL; mean (SD)	7.82 (0.72)	7.96 (0.76)	7.94 (0.76)
ALT, ULN; mean (SD)	4.86 (2.29)	4.44 (2.09)	4.53 (2.13)
HBV Genotype			
B	68 (53.13)	194 (37.31)	262 (40.43)
C	59 (46.09)	318 (61.15)	377 (58.18)
Others	1 (0.78)	7 (1.34)	8 (1.23)
NA	0 (0)	1 (0.19)	1 (0.15)

ALT, alanine aminotransferase; CR, complete response; HBeAg, hepatitis B e antigen; HBsAg, hepatitis B surface antigen; HBV, hepatitis B virus; NCR, noncomplete response; SD, standard deviation; ULN, the upper limit of normal.

**Table 3:** Association of carnitine palmitoyltransferase 2 (*CPT2*) genotypes with complete response.

*CPT2* (SNP rs1799821)	CR (n,%)	NCR (n,%)	OR (95% CI)	*P*	AOR^*^ (95%CI)	*P*
AA	52 (40.63)	280 (53.85)	Referent	-	Referent	-
*Genotypic model*						
GG *vs*. AA	10 (7.81)	30 (5.77)	1.80 (0.83-3.90)	0.14	1.67 (0.74-3.75)	0.21
GA *vs*. AA	66 (51.56)	210 (40.38)	1.71 (1.14-2.57)	9.78×10^-3^	1.70 (1.12-2.59)	0.01
*Dominant model*						
(GG/GA) *vs*. AA	76 (59.38)	240 (46.15)	1.72 (1.16-2.56)	7.01×10^-3^	1.70 (1.13-2.55)	0.01

^*^Gender, Age, HBV genotype, baseline HBV DNA, baseline HBsAg, baseline HBeAg, and baseline ALT were adjusted in the multiple logistic regression analyses.

AOR, adjusted odds ratio; CI, confidence interval; CR, complete response; NCR, noncomplete response; OR, odds ratio.

## Discussion

Achievement of HBsAg loss represents the hallmark for a functional cure of chronic HBV infection. Currently, there are no effective antivirus drugs that directly target HBsAg clearance. Functional T cells (with helper or cytotoxic effects) and antibody-producing B cells benefit HBV control. In recent years, immunometabolism opens new therapeutic possibilities for modulation of immune responses during infections and inflammatory diseases. In this study, we primarily investigated the immunomodulatory effect of L-Cn on immune cells in chronic HBV infection.

L-Cn is essential to transport the chains of fatty acids into the mitochondria for β oxidation. Manipulation of cell metabolic programs dictates T cell function and differentiation (10, 22). It has been reported that the serum L-Cn levels were stepwise altered in HBV-cirrhosis and HCC, and plasma L-Cn levels remain lower in patients with HBsAg loss after Peg-IFNα based treatment (12, 23), suggesting L-Cn plays a critical role in affecting the outcome of chronic HBV infection. The present study revealed a significant elevation of L-Cn in patients with chronic HBV infection and HBV-HCC. Of note, plasma L-Cn levels were positively correlated with plasma levels of ALT, AST, and bilirubin. Based on the finding that carnitine concentration within the liver is 25-50 times higher than that of plasma levels (11), we assumed that L-Cn might be derived from the leakage of hepatic carnitine; this hypothesis should be elucidated in further studies.

According to previously published data, the seemingly high doses of L-Cn demonstrating pharmacological activity without toxicity were selected for *in vitro* assays (14, 24). In the present study, we demonstrated that L-Cn stimulation significantly decreased T and B cell proliferation, IFN-γ-secreting and IL-21-producing T cells, and IgG-producing B cells. Previous publications have demonstrated the favorable effect of CD4^+^ helper T cells in facilitating HBV-specific CD8^+^ T cell response and enhancing HBV-related antibody production from B cells through IL-21 (25–27). These results suggest that L-Cn may affect HBV control by downregulating cellular and humoral immune responses. In contrast, L-Cn stimulation generated much more IL-10-producing Treg cells and simultaneously up-regulated multiple inhibitory receptors on T cells, indicating an inhibitory effect on T cell response. It has been verified that inhibitory molecules, such as PD1, CTLA4, Tim3, and 2B4, are upregulated on exhausted HBV-specific T cells, which contribute to HBV persistence (28–30). Moreover, we provide evidence that L-Cn administration altered the transcriptional profiles of immune cells and affected multiple core processes. Furthermore, we genotyped some SNPs that were reported to be associated with L-Cn levels (31–33). Of note, we confirmed that SNP rs1799821 was strongly associated with plasma L-Cn levels as reported. An interesting observation was that patients who carried the rs1799821 G allele showed lower levels of plasma L-Cn and was associated with the complete response following Peg-IFNα therapy. Thus, it is reasonable to speculate that the immunosuppressive effect of L-Cn might be related to the failure of HBV control; reprogramming L-Cn metabolism may serve as a potential therapeutic target for HBV infection. Further experiments are needed to clarify this hypothesis and the mechanisms underlying the immunoregulatory function of L-Cn.

This study also showed that plasma L-Cn levels did not vary with age and sex (**Supplementary Figures 2A, B**). It should be noted that around 75% L-Cn comes from diet and only 25% comes from endogenous synthesis with lysine and methionine in the kidney, liver, and brain; therefore, it is not surprising that L-Cn was dynamically regulated by dietary exposure (11). One month’s red meat diet consumption had been shown a significant increase in plasma carnitine levels, relative to either non-meat or white meat diets (34). Interestingly, diet intervention seems to affect plasma L-Cn levels. Consistently, we found an increase of plasma L-Cn levels following one week of the beef-rich diet; however, short-term beef provocation did not cause plasma L-Cn levels fluctuation, which suggested L-Cn as a potential stable plasma biochemical marker (**Supplementary Figures 2C, D**). Thus, it is reasonable to speculate that shifting diet habits to create a low plasma L-Cn condition may improve the antiviral immune response and favor the clinical outcome of chronic HBV infection. Because almost all Chinese are omnivores, no vegetarian or strict vegetarian is enrolled for the L-Cn challenge test; further experiments are needed to validate. Importantly, a study showed intestinal microbiota metabolism of dietary L-Cn to produce trimethylamine-N-oxide (TMAO), which accelerated the development of atherosclerosis (35). Furthermore, the study demonstrated that the transformation of γ-butyrobetaine into TMA/TMAO by gut microbiota was induced by omnivorous dietary patterns and chronic L-Cn exposure (36). Interestingly, an increasing number of studies have shown that the intestinal microbiota regulates host metabolism and immune homeostasis in chronic HBV infection (37–39). However, the link between L-Cn, gut microbiota, and the risk of HBV-related liver disease remains largely unknown and should be elucidated in further studies.

There are several limitations of this study. Due to the limited availability of clinical specimens, especially from HBV-HCC and patients who achieved HBsAg loss, future studies with more cases are needed to verify our findings further. Ideally, the correlation between plasma L-Cn levels and complete response should be stronger confirmed in the further longitudinal cohort study. Additionally, the RNA-sequencing of L-Cn-treated PBMCs and the GSE62595 dataset can only show the transcriptome changes at the overall level, further studies on exploring the modulation mechanism of L-Cn on a single set of cells should be taken to dissect the related signaling pathway. Moreover, the dose of L-Cn used in this study is over the range of physiological condition, the immunomodulatory effect of L-Cn on immune cells should be evaluated *via in vivo* experiments with the HBV mouse model. Finally, it remains unknown if diet intervention could downregulate plasma L-Cn levels effectively. More healthy controls and patients with HBV infection should be recruited to perform a much more extended time diet intervention to confirm the conclusion.

In summary, the present findings demonstrated that an elevated plasma L-Cn might precipitate the persistence of HBV infection by exerting an immunosuppressive function, indicating a beneficial effect of low L-Cn level in chronic HBV infection. Management strategy targeting L-Cn or shifting diet habits to less L-Cn intake might serve as a potential therapeutic intervention in HBV infection.

## Data Availability Statement

The datasets presented in this study can be found in online repositories. The names of the repository/repositories and accession number(s) can be found below: https://www.ncbi.nlm.nih.gov/sra/PRJNA673447.

## Ethics Statement 

This study involving human participants were reviewed and approved by the Ethical Committee of Nanfang Hospital (Guangzhou, China). The patients/participants provided their written informed consent to participate in this study.

## Author Contributions

SG and YL designed the study. SG, XF, GY, CC, SZ, and XL performed the experiments and analyses. SG and XF collected samples and laboratory data. HC and DJ performed analyses. SG, YL, and LT wrote the manuscript. JH and YL supervised the study. All authors contributed to the article and approved the submitted version.

## Funding

This work was supported by grants from the National Natural Science Foundation of China (81971933, 81770592, and 81671570), National Science and Technology Major Project of China (2017ZX10202202-004 and 2018ZX10301202).

## Conflict of Interest

The authors declare that the research was conducted in the absence of any commercial or financial relationships that could be construed as a potential conflict of interest.

## Supplementary Material

Supplementary Figure 1 No association of plasma L-Cn levels with the indicated SNPs.

Supplementary Figure 2 Diet intervention influences plasma L-Cn levels. **(A, B)** No correlation between the plasma L-Cn levels and age, and no significant change in plasma L-Cn level between males and females in subgroups. **(C, D)** Schedules of human L-Cn challenge test. Eight healthy volunteers were recruited and the dynamic L-Cn levels were recorded. To evaluate the influence of diet intervention on plasma L-Cn levels, two male participants fasted overnight (10 h) before performing the short-term L-Cn challenge test, which included a natural source of L-Cn (240 g cooked-beef, a major source of dietary L-Cn, equivalent to an estimated 180 mg L-Cn), and venous blood samples were collected at indicated time points for L-Cn detection. After an overnight fast, subjects were challenged with 240 g cooked-beef (a major source of dietary L-Cn, be equivalent to an estimated 180 mg L-Cn). An additional one-week dietary intervention L-Cn challenge test was designed to examine the impact of dietary beef on L-Cn among another healthy adult participants (n=6, all omnivores, 3 males and 3 females, with normal renal function; age: minimum 23 years, median 28 years, and maximum 40 years; body mass index (BMI): minimum 18.60, median 20.13, and maximum 26.22). After one month of their habitual diet, which was designed to reflect serum L-Cn fluctuation, the volunteers were shifted to a weeklong beef-rich diet (around 220 g cooked beef during each lunch and dinner, equivalent to an estimated 330 mg L-Cn per day). **(A)** Spearman’s rank correlation test. **(B)** Mann-Whitney *U* test. **(D)** Friedman test and Dunn’s multiple comparisons test. **P* < 0.05. L-Cn, L-carnitine.
