# Supplementary figures and images for: High L-Carnitine Levels Impede Viral Control in Chronic Hepatitis B Virus Infection
Source: Front Immunol. 2021 Jun 21;12:649197. doi: 10.3389/fimmu.2021.649197 (PMC8255973; doi:10.3389/fimmu.2021.649197)

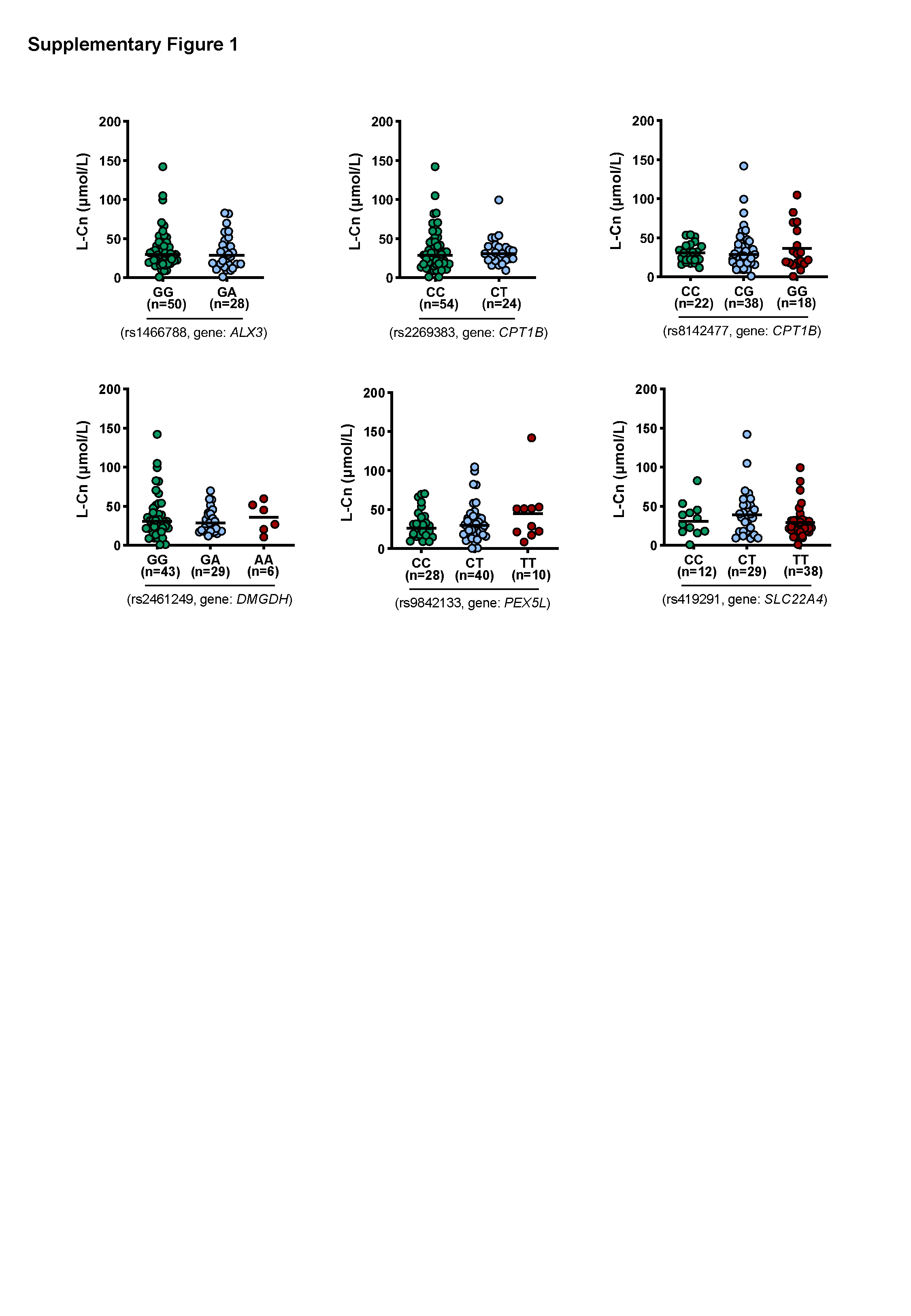

Supplement: Supplementary Figure 1 — No association of plasma L-Cn levels with the indicated SNPs. [file Image_1.tiff]

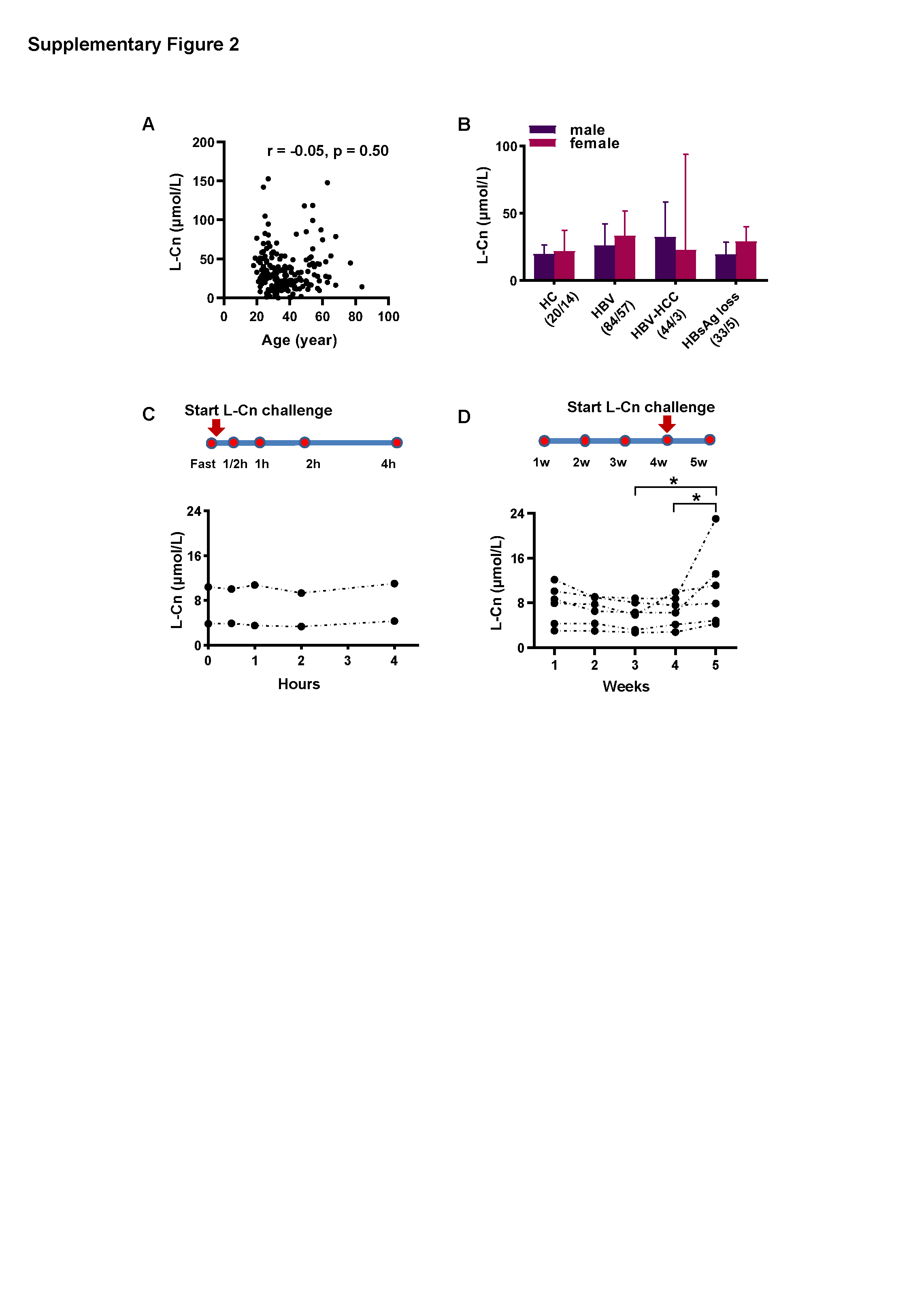

Supplement: Supplementary Figure 2 — Diet intervention influences plasma L-Cn levels. (A, B) No correlation between the plasma L-Cn levels and age, and no significant change in plasma L-Cn level between males and females in subgroups. (C, D) Schedules of human L-Cn challenge test. Eight healthy volunteers were recruited and the dynamic L-Cn levels were recorded. To evaluate the influence of diet intervention on plasma L-Cn levels, two male participants fasted overnight (10 h) before performing the short-term L-Cn challenge test, which included a natural source of L-Cn (240 g cooked-beef, a major source of dietary L-Cn, equivalent to an estimated 180 mg L-Cn), and venous blood samples were collected at indicated time points for L-Cn detection. After an overnight fast, subjects were challenged with 240 g cooked-beef (a major source of dietary L-Cn, be equivalent to an estimated 180 mg L-Cn). An additional one-week dietary intervention L-Cn challenge test was designed to examine the impact of dietary beef on L-Cn among another healthy adult participants (n=6, all omnivores, 3 males and 3 females, with normal renal function; age: minimum 23 years, median 28 years, and maximum 40 years; body mass index (BMI): minimum 18.60, median 20.13, and maximum 26.22). After one month of their habitual diet, which was designed to reflect serum L-Cn fluctuation, the volunteers were shifted to a weeklong beef-rich diet (around 220 g cooked beef during each lunch and dinner, equivalent to an estimated 330 mg L-Cn per day). (A) Spearman’s rank correlation test. (B) Mann-Whitney U test. (D) Friedman test and Dunn’s multiple comparisons test. *P < 0.05. L-Cn, L-carnitine. [file Image_2.tiff]
